# Role of pharmacogenomics in drug discovery and development

**DOI:** 10.4103/0253-7613.43158

**Published:** 2008-08

**Authors:** A. Surendiran, S.C. Pradhan, C. Adithan

**Affiliations:** Department of Pharmacology, JIPMER, Puducherry, India

**Keywords:** Drug, genotype, pharmaceuticals, pharmacogenetics, pharmacogenomics

## Abstract

Pharmacogenetics and pharmacogenomics are two major emerging trends in medical sciences, which influence the success of drug development and therapeutics. In current times, though pharmacogenetic studies are being done extensively for research, its application for drug development needs to get started on a large scale. The major determinants of success of a new drug compound, viz safety and efficacy, have become more predictable, with the advent of pharmacogenetic studies. There is a need felt for pharmacogenomic studies, where the effects of multiple genes are assessed with the study of entire genome.

Pharmacogenetic studies can be used at various stages of drug development. The effect of drug target polymorphisms on drug response can be assessed and identified. In clinical studies, pharmacogenetic tests can be used for stratification of patients based on their genotype, which corresponds to their metabolizing capacity. This prevents the occurrence of severe adverse drug reactions and helps in better outcome of clinical trials. This can also reduce attrition of drug compounds. Further, the variations in drug response can be better studied with the wider application of pharmacogenomic methods like genome wide scans, haplotype analysis and candidate gene approaches. The cost of pharmacogenetic testing has become very low, with the advent of newer high throughput genotyping systems. However, the cost of pharmacogenomic methods continues to be very high. As the treatment with several drugs is being more and more pharmacogeneticaly guided (e.g. warfarin and irinotecan), the FDA has laid down guidelines for pharmaceutical firms regarding submission of pharmacogenetic data for their drug products in labelling.

## Introduction

In the process of drug discovery, most drug compounds that act on the target during the early phases fail to emerge as therapeutic agents. This could be due to unexpected failure of efficacy or occurrence of adverse events. Pharmacogenetics involves the study of single gene mutations and their effect on drug response. The term pharmacogenomics is much broader and it involves surveying the entire genome to assess several determinants of drug responses.[1] The current status of pharmacogenomics is restricted to the areas of medical research and epidemiological studies.

Many treatment regimens like that of oral anticoagulants and cancer chemotherapy are now guided by the pharmacogenetic status of the patient, to avoid toxicity as well as treatment failures.[2,3] The traditional method of trial and error in selecting a drug dose is gradually being replaced by pharmacogenetic methods. Pharmacogenetics, which is being utilised currently in therapeutics, can also be utilised in drug discovery and development.

## Genetic Factors in Drug Effects

The genetic factors that can affect the drug response include variations in the genes coding for drug metabolizing enzymes, receptors and transporters. A classic example of genetic polymorphism affecting drug metabolism would be that of the enzyme CYP2C9, which is coded by the polymorphic gene *CYP2C9*. Its variant alleles namely *2 and*3 are poor metabolizers, with only 12% and 5% respectively of enzyme activity, as compared to normal allele *1.[4] Variations in drug response due to polymorphism of receptors can be illustrated with β_2_ adrenergic receptor polymorphisms, where homozygous mutants with decreased expression of β2 adrenergic receptors do not have a predictable response to the use of drugs like salbutamol in asthma.[5,6] Similarly, polymorphisms of transporters affect drug effects. The promoter region of the serotonin transporter gene exists in two forms: short form (sl and ss) and long form (ll). The short form of this polymorphism (ss) is associated with decreased clinical response to citalopram in children or adolescents with depression or anxiety.[7,8] The variant allele *5HTTLPR-S* coexisting with the CYP2C9*3 variant allele is found to be associated with an even greater risk of major depressive disorder.[9]

Depending upon the nature of the drug being tested, these genetic variations can cause either toxicity or failure of treatment in a clinical trial. Pharmacogenetic methods can be used to identify these genetic factors.[10,11] When a large number of patients are to be genotyped, as in a clinical trial, high throughput genotyping methods like Amplifluor^®^ and TaqMan ®[12] can be used at a reasonable cost. Methods like haplotype mapping, where more than one genetic variation in a single chromosome is studied together, can identify the genetic basis of diseases, as well as newer targets for drug development.[5]

## Current Scenario in Drug Discovery and Development

### Time factor in drug development

Drug discovery and development is an elaborate and time consuming process. The average time taken from synthesis of a new chemical entity (NCE) to its marketing has increased from an average of 7.9 years in 1960 to an average of about 9 to 12 years in 1990.[13] It includes two major phases, phase I being the discovery of the drug compound, which is the most crucial stage in drug development, and phase II being animal and clinical studies followed by marketing. The increase in time duration can be attributed to the complexity of the clinical trials and rigid regulations. Out of five thousand compounds evaluated initially during the drug screening process, only five of them enter clinical trials. Of these five drugs, only one gets approved for marketing. 

The process of drug development includes pathway identification and target selection, screening of chemical compounds, drug development, preclinical and clinical studies and, finally, drug marketing.

### Cost factor in drug development

The total expenditure involved in a drug development and its launch is very high. A study on the cost of drug development done by DiMasi *et al.*, in 2003, gave an estimate of US $ 802 million in the year 2000, for an NCE development, right up to its marketing. This indicates the resource cost for a firm, and not the effective cost. The capital investment has also been addressed by DiMasi *et al.* in this study. The estimated capitalised phase cost for an NCE was given as US $ 1.6 million for animal model testing, 15.2 million for phase I trial, 16.7 million for phase II and US $ 27.1 million for phase III trials.[14] In a majority of the cases, the termination of a clinical trial occurs during phase II and phase III and the loss of financial resources are high, since millions of dollars have already been utilised. DiMasi has shown that by reducing the clinical phase duration and increasing the success rates, the expenditure for development per NCE is also reduced.[13]

### Implications of termination of a clinical trial

**Figure 1:**
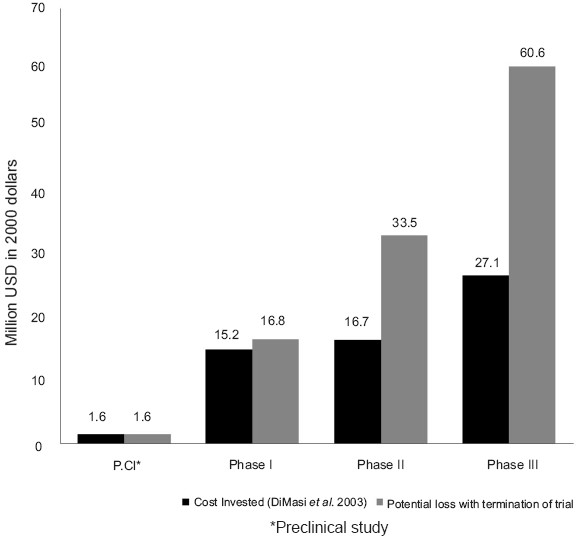
Potential financial loss with premature termination of a clinical trial

The most common reason for termination of a clinical trial or failure of a compound is lack of efficacy, which is followed by safety concerns.[1] This occurs mostly in phase II and phase III of clinical trials. Phase III study, being done on a larger scale and on a larger study population, utilises a very large share of the resources. When a trial is thus ended, the loss incurred financially as well as in terms of time is unacceptable for a pharmaceutical firm. Figure 1 shows the financial losses incurred by termination of clinical trial in each phase.

Before the advent of pharmacogenetic studies and proteomics, efficacy and safety concerns were poorly predictable in a clinical trial. In current times, the predictability of safety and efficacy of a drug has increased to a significant level, as both are influenced by the genetic status of the individual, which can be assessed by pharmacogenetic studies. 

## Pharmacogenomics and its Application in Drug Development

The applications of pharmacogenomics in drug development process are briefly given in Table 1. It can be seen that it has a vital role at every key stage of the drug development process.

**Table 1:** Application of pharmacogenetic/pharmacogenomic methods in various stages of drug development

*Stage*		*Application of pharmacogenetics/pharmacogenomics*
Drug target Identification		Identification and characterisation of the gene coding for the drug target and to assess the variability
Phase I clinical trial		Patient selection – Inclusion/Exclusion criteria
		Dose range selection
Phase II clinical trial		Dose modification
Phase III clinical trial		Interpretation of trial results based on pharmacogenetic test results
Phase IV clinical trial		Analysis of reported adverse events with pharmacogenetic tests
Regulatory issues		Requirements for submission of pharmacogenetic data during development by FDA
Patient therapeutics		Personalization of drug therapy
		Pharmacogenetic data in drug labelling
		Identification of responders and non responders
		Identification of high risk groups of adverse events

### Drug target and pharmacogenomics

The process of drug discovery starts with the identification of a potential target at which the drug can act. The target can be an enzyme in a vital pathway, a receptor, a transporter, a protein in signal transduction or any protein produced in a pathological condition. After sequencing of the human genome, the number of drug targets was estimated to be around 8000, out of which 4990 could be actually acted upon - 2329 for antibodies and 794 for drug proteins.[15] Based on ligand binding study, 399 molecular targets belonging to 130 protein families have been identified.[16] However, currently, the number of targets ranges only around 218, though the estimated numbers are very high.[17] These targets are known to exhibit variations owing to genetic polymorphisms. Drugs which are based on targets showing wide polymorphisms can have variations in their effect. For example, polymorphism of β2 adrenoceptor gene, as discussed earlier, has produced responders and non responder phenotypes.[7] This can lead to inconsistent results in the preclinical and clinical studies that would follow if such a drug compound is pursued. Such targets can be avoided for drug compounds and other suitable targets can be selected. Thus, at an early stage, the targets can be characterised based on pharmacogenetic studies combined with proteomics and suitable drug compounds selected for further investment.

In most cases, variation in drug response in a disease is attributed to many genes rather than a single gene mutation. The results of pharmacogenetic study do not apply when used clinically, as only single gene mutations are studied when in fact multiple genes are involved. In such cases, more than pharmacogenetic study, it would be appropriate to do pharmacogenomic study comparing single nucleotide polymorphism (SNP) maps and gene expression between normal and affected individuals. This can identify the genetic factors associated with the disease and thus provide newer targets to characterise and evaluate, for the purpose of drug development. Those that could be potential future drug targets can be called as “tractable” or “drugable” targets.[1] With the availability of advanced human genome sequences, the genes can now be analyzed *in silico*  for coding regions of the tractable targets. Polymorphisms of P2Y_12_  receptors in platelets have been identified to be associated with increased risk of coronary artery disease by haplotype analysis. In the future, this can be a potential target for a drug compound produced against coronary artery disease.[18] Selection of the right target for development of a drug is vital and pharmacogenomics can play a vital role in it.

### Pharmacogenomics and clinical trials

Pharmacogenomics in clinical trials is a relatively new area in which considerable hesitation is shown by pharmaceutical companies. Incorporation of pharmacogenomic testing with clinical trials has multiple advantages.

The two most important concerns for new drug development are efficacy and safety. Before the advent of pharmacogenetic tools, the predictability of both these factors was very low. This translated into heavy financial loss due to attrition of the drug compound during clinical trials. In current times, the scenario has changed and with the availability of sophisticated pharmacogenetic tools, the attrition rate can be significantly reduced. This translates into reduction in loss of financial resources for drug development. With *in vitro*  methods, it can be identified during preclinical studies, if the drug is metabolized by polymorphic enzymes,[19] and a decision regarding continuation of the trial can be made. Also, this information can help in selecting appropriate patients with normal metabolizing enzymes in phase I clinical trial; it can also help prevent adverse events.[20] It must be noted that pharmacogenetic principles can be used for inclusion or exclusion criteria only when the metabolic pathway of the drug is known. In cases of exploratory studies, where knowledge regarding the metabolism of the drug is not known, pharmacogenetic principles cannot be applied for selection of subjects in the early phases of studies. However, the acquisition of pharmacogenetic data in the early phases of clinical trial can be useful for the later phases.

### Prediction of efficacy of drug

In contrast to the conventional methods where preclinical and clinical studies are done with the purpose of determining efficacy, drugs designed with pharmacogenomic support have a predetermined efficacy status. The chance of a drug failing in preclinical and clinical studies due to the absence of efficacy is minimized. The efficacy of a drug, to a great extent, is determined by appropriate target selection, which can be guided by pharmacogenomic methods. To cite an example, the drug trastuzumab, an anti-HER2 monoclonal antibody against metastatic breast cancer, was found to be effective only in women who were over expressing the HER2 protein during early clinical trials. In the subsequent trials, studies were done only on women found to be over expressing the HER2 protein.[21,22] The drug got approval for marketing with the requirement of testing for HER2 over expression, before starting therapy. Had this drug been tested in a whole population without genetic stratification, the efficacy of the drug would not have been brought out. 

Pharmacogenomics can also be used to identify the target population that would benefit the most from the drug. A typical example would be association between polymorphisms in the apolipoprotein E (APOE), cholesteryl ester transfer protein CETP, stromelysin-1 and β-fibrinogen with progression of atherosclerosis, cardiovascular events and death. It was shown that people with such polymorphisms derived the maximum benefit from HMG-CoA inhibitors, as compared to those without the polymorphisms.[23–26]

### Prediction of safety of drug

The second major concern in a clinical trial is the safety profile of the drug. Data provided by preclinical studies can be applied to humans only to a certain extent. During a clinical trial, the occurrence of a serious adverse event could jeopardize the drug status. Usually, such an event would culminate in the termination of the trial.

Drug toxicity occurs mainly due to increased plasma levels of the drug, which may be the result of poor metabolizing capacity owing to genetic polymorphisms. Many of the genes (*CYP2C9, CYP2C19, CYP2D6*, etc) have been intensely studied in various populations and characterized for various drug groups. The role of pharmacogenetics in predicting the efficacy of most drugs is limited, as they are usually dependent on more than one gene. In contrast, the safety of many drugs is, to a great extent, determined by the plasma drug levels, which in turn depends upon the drug metabolizing capacity. The drug metabolizing enzymes have been identified to exhibit single nucleotide polymorphisms and hence pharmacogenetic approach has a better predictability of the safety of a drug.

The cost of genotyping for SNPs has come down drastically. The estimated cost for analyzing 1000 DNA samples for a single marker for SNP is 300 USD. This amounts to 0.3 USD per genotype.[27] With the availability of high throughput genotyping methods, pharmacogenetic testing can be incorporated into inclusion criteria for selecting a subject for the trial. Those who are identified as poor metabolizers of the drug tend to attain a higher plasma concentration of the drug, and hence the higher incidence of toxicity. When poor metabolizers are avoided in the study, the occurrence of serious adverse events is reduced. For example, polymorphisms of *UGT1A1 * determines the toxicity response to irinotecan due to its effect on the plasma levels of irinotecan.[28] Thus, the study population can be stratified into groups based on their drug metabolizing capacity and those with diminished capacity can be avoided in the study or given lower doses of the drug. The drugs can be thus marketed with pharmacogenetic details along with the product.

### Concerns with pharmacogenomics in drug development protocols

When drugs are tested, based on pharmacogenetic profile of patients and their categorization, it gives the impression that those with poor metabolizing capacity are being left out. Seen in a broader aspect, it may be noted that this method only brings to light what has not been realized in the pregenomic period, where the occurrence of serious adverse events in clinical trials and in clinical practice has been poorly explained. By utilizing pharmacogenetic tools and understanding the cause for adverse effects, the drug induced morbidity in poor metabolizers can actually be prevented when pharmacogenetics gets linked with clinical practice and drugs are prescribed with appropriately guided doses. It must also be realized that the subset of population with poor metabolizing capacity, arising from genetic polymorphism, constitutes only a minor segment, with very low frequency distribution. In the event of an enzyme polymorphism occurring in a larger population, the development of such a drug is avoided by the pharmaceutical firm.

Another concern would be the cost that the patient would have to endure for pharmacogenetic testing before starting therapy, as the drug has been approved for marketing, based on pharmacogenetic results. The cost of genotyping for single nucleotide polymorphisms may not be affordable in many developing and underdeveloped countries. However, with advancements in technology, this price may come down in the near future. As mentioned earlier, the cost for genotyping 1000 DNA samples would be at the rate of 0.3 USD per genotype. But when the cost is calculated for a single patient sample, it amounts to more than 130 USD, as the price for assay marker setup is fixed.[27] Thus it seems, genotyping is cost effective only if it is done on a larger scale, which would be the case if it is used for therapeutic testing.

### Pharmacogenetics in patient care

The most commonly applied pharmacogenetic testing for patient care is detection of polymorphisms of genes coding for drug metabolizing enzymes and assisting in dosage selection or modification. A recent study has shown that 59% of the drugs that cause adverse effects are metabolized by polymorphic enzymes.[29] There have been reports of accentuated action of warfarin in patients with increase in INR (International Normalized Ratio) at normal doses and on subsequent pharmacogenetic testing, they were found to be poor metabolizers with *3/*4 and *1/*3 polymorphisms of *CYP2C9*.[2,30,31] As a result of significant association between *CYP2C9* polymorphisms and warfarin toxicity, the FDA has approved inclusion of pharmacogenetic data in the product label for warfarin and the warfarin product label now carries the genetic information.[32] This makes warfarin therapy safer during initiation and maintenance of treatment. 

Another potential use of pharmacogenetics is identification of population with risk factors not directly related to the drug. It has been found that patients with prothrombin gene mutation have a risk of developing cerebral vein thrombosis and when these patients are on oral contraceptive pills, they run an even higher risk.[33] This information, when made available, could prevent the occurrence of adverse events with oral contraceptives in high risk patients having a genetic susceptibility. The reduction in adverse effects reflects upon the safety profile of the drug and in its marketing value. There is always a persistent hesitation among pharmaceutical industries to base their drug products on pharmacogenetic testing. This is due to various factors like segmentation of the market based on genetic profile and increase in the product and health care costs. These factors keep the pharmaceutical industry in a “wait and see” stance for producing pharmacogenetic data for their products.

### Challenges in inclusion of pharmacogenetics in a clinical setting

The inclusion of pharmacogenetic data in health care delivery poses many more challenges. The knowledge regarding the influence of genetics on specific drug response and dose modulation is yet to be imparted to many of the clinicians. This is, in fact, a more complicated and challenging issue. The drug dosage is likely to become complicated with the involvement of genetic data. Moreover, the fact that most of the patients receive more than one drug for a single disease and that many combination therapies exist are likely to make the task enormous. Adjustment of drug dosage based on clinical parameters like liver and renal function test is well accepted and practiced. However, there is hesitation among clinicians to modulate the dose on the basis of the genetic status of the individual and they prefer to rely on the clinical parameters. This could be due to the hesitation to give up the “trial and error” method or unfamiliarity with the genetic principles of drug response.[34]

### Drug response variability and pharmacogenomics

The factors affecting the drug response for a disease are multiple. They can be categorized as unpredictable (environmental influence, compliance and genetic profile of the patient) or predictable factors (age, sex, race, body weight, disease state, nutritional status etc). With the advent of pharmacogenetic testing and the human genome breakthrough, the genetic makeup of the patient can now be considered a predictable factor for drug response. However, the predictability based on pharmacogenomic testing may not be absolute, as there are other unpredictable factors also. Yet, with the application of pharmacogenomic testing, the drug can be made relatively more efficacious and safer. The scope of determining drug response variability based on pharmacogenetic testing is very limited, as compared to pharmacogenomics. A study done on the polymorphisms of promoter and coding regions of β_2_ adrenoceptors has shown association between the haplotypes and bronchodilator response to salbutamol, whereas no association was seen between individual genotypes of b_2_ adrenoceptor and bronchodilator response.[35] Another example would be the variation in drug response to ACE inhibitors. Different studies have shown different association between the genotypes ACE *I/I*, ACE *I/D* and ACE *D/D* and response to ACE inhibitors.[36] As per the study done by Cannella *et al.* (1998), the genotype of ACE does not predict the effectiveness of ACE inhibitor therapy in reducing the left ventricular mass in chronic uremic patients.[37] Hernandez *et al.* (2000) have shown a significant reduction in left ventricular mass index in renal transplant patients with LVH on treatment with lisinopril with ACE *D/D* genotype.[38] Kohno *et al.* (1999) have shown that patients with ACE *D/D* genotype are less likely to have regression of LVH, when treated with ACE inhibitors.[36] Thus, a broader study involving multiple genes or allele variants is needed to understand the intricacies of drug response variability and to identify the factors affecting it. The methods which can be used for this purpose are candidate gene approach, genome wide scan approach and haplotypes analysis.

## Methods of Studying Genetic basis of Drug Response Variations

### Candidate gene approach

Candidate gene approach for identifying genetic determinants of drug response variability involves identifying association between various allelic variants or SNPs within the candidate gene and the drug response.[39] The method of candidate gene approach starts with identification of candidate genes. For a drug response, candidate genes could be the genes coding for the drug metabolizing enzyme, the proteins involved in the drug transport, the proteins involved in the cellular mechanisms, the receptor proteins etc. The candidate gene is studied for allelic variants. A candidate gene can have more than one allelic variant or SNP. Candidate gene effects can be studied in case (people with altered drug response) and controls (people with normal drug response). Candidate gene studies are less expensive than linkage disequilibrium studies and genome wide scans.[39] A limitation of candidate gene studies is that spurious association can occur if the case and controls are not adequately matched. Further, variants in other genes, which are remotely influencing the drug response, cannot be identified. Moreover, a level of understanding of the drug response pathway is required to identify the candidate genes unlike genome wide scans.

### Genome wide scan

Genome wide scan is a very extensive and elaborate study method for effects of various allelic variants occurring throughout the genome and the drug response in a disease condition. This method involves identification of all the allelic variants in the entire human genome and the creation of an SNP map. This is tested for association with drug response variation.[40] The advantage of genome wide scan approach, as compared with the other methods, is that, it can identify the polygenic determinants of drug response. In contrast to candidate gene approach, genome wide scan approach does not require prior information regarding the drug response pathway in question. This approach increases the translation level of genetic testing in clinical response. It is estimated that the human genome has approximately three million SNPs and it is not cost effective to screen all the SNPs. As a result, only representative SNPs that are distributed evenly across the genome are selected for testing. This may be in the range of 200000 to 300000 SNPs per human genome.[41] The SNP maps can be studied for linkage disequilibrium and disease or drug response associations. The disadvantages of genome wide scan approach is that it is very expensive to perform and since a large number of SNPs are mapped, they need to be further evaluated, as it is not hypothesis driven like that of the candidate gene approach.

### Haplotype analysis

Haplotype analysis for drug response variation involves the study of clusters of SNPs occurring in linkage disequilibrium in a chromosome and their association with the drug response. This is different from genome wide scan approach in that only selected haplotypes are analyzed and not the entire genome. Haplotype blocks are created by clustering selective SNPs and their linkage disequilibrium is tested with family studies. The haplotype blocks are then tested for association with clinical outcomes. 

Haplotype analysis provides more information than pharmacogenetic study of single nucleotide polymorphisms and is cost effective. Based on these studies, the various genetic determinants of a drug response can be identified and drug development can be customized accordingly. The same methods can be used in clinical trials, to identify high risk groups for adverse effects and to derive an explanation for the outliers in the study group for lack of efficacy.

### Pharmacogenomic data in labeling and their limitations

Pharmacogenomic data may be integrated in the labeling of a product. Such data might be useful in identifying patients who require dose adjustments for the prevention of adverse effects or lack of efficacy. The FDA has approved inclusion of pharmacogenetic data in the labelling of drugs like warfarin[32] and irinotecan,[42] mainly for safety concerns. The inclusion of pharmacogenetic data for improving the efficacy of a drug is a difficult task to achieve, owing to reasons like low predictability with pharmacogenetic testing involving single gene mutations and the potential compromise of marketing for the product. However, this change in labelling can be productive only if there are genotyping facilities available, which is yet to happen in many developing countries. It is, however, recommended by the FDA that while labelling with pharmacogenetic data, the industry should also develop methods of pharmacogenetic testing for that biomarker and give complete information about the test and its interpretation, and the dose modulation.[43]

Although the FDA encourages inclusion of pharmacogenetic data in product labels, there are certain limitations of application of pharmacogenetic methods. The variability in the drug response due to non genetic factors cannot be predicted. Data obtained by genetic testing must be taken only with the limitation that there are also other factors determining the response. 

Ethnic diversity of human population has made it necessary for pharmacogenetic testing to be done in every ethnic group for the product to be utilized by that group.[44] In addition to pharmacogenetic data of various ethnic groups, it is required that there are pharmacogenetic testing centers in countries where the drug is intended to be marketed. This also requires that a standard pharmacogenetic testing method is adopted uniformly to avoid variation in test results. The cost of testing must be substantially low for the general public. Moreover, the extent of usage of product labels by clinicians is insufficient to make an impact on patient therapeutics. Clinicians may hesitate to use a product that requires pharmacogenetic testing, as it would incur additional cost for the patient. This highlights the need for imparting relevant knowledge to the clinicians on how to modulate therapeutics based on pharmacogenetic data. Until this goal is achieved, a pharmaceutical firm would restrain itself from using pharmacogenomics in drug development.

### FDA, Pharmacogenomics and drug development

The FDA has provided certain guidelines for submission of pharmacogenetic test data by the pharmaceutical industries, as a part of drug development.[43] As most of the pharmacogenetic test results are not well-established scientifically, such studies cannot be used by the FDA for regulatory decisions. Currently, the FDA has made submission of pharmacogenetic data mandatory for specific cases and supports voluntary submission for other specific cases, which are described below. Guidelines have also been released pertaining to the time when a complete pharmacogenetic report is to be submitted and when an abbreviated report is to be submitted. Further, there are separate guidelines for submission of pharmacogenetic data for investigational new drug applications and unapproved and approved marketing applications. However, even though the FDA is of the opinion that pharmacogenomic integration in drug development is advantageous for all sectors, drug industries tend to have a difference of opinion among themselves on this issue.[45]

As mentioned above, a well-established pharmacogenetic test is required as a valid biomarker for making regulatory decisions by the FDA. For a pharmacogenetic test to be accepted as a valid biomarker, the test should have a sound scientific framework and well established characteristics. An example for a valid biomarker in pharmacogenetic tests would be those for drug metabolizing enzymes as a marker for drug efficacy and safety. Patients with variant alleles of the gene CYP2C9 and VKORC1 require lower doses of warfarin, as compared to patients with normal wild type alleles. This is a valid biomarker and pharmacogenetic data has been incorporated in the drug label for warfarin.[2,30,32]

The FDA has made submission of a complete pharmacogenetic data report mandatory, if these results have been used for decision making in the animal study, to support the safety of the drug, or in clinical trials, for the selection of subjects, dose range or its modification. The complete data is also required in cases where the sponsor uses the pharmacogenetic test results to validate safety, efficacy, dosage selection and mechanism of action in the clinical trials. However, in cases where such pharmacogenetic test results are not being used by the sponsor to support the results of the trial, but the test is a valid biomarker for that drug, an abbreviated report of the pharmacogenetic test has to be submitted to the FDA. In cases where the pharmacogenetic testing has been done as an exploratory study or for research, it is not mandatory to submit such data, as they cannot be considered as valid biomarkers. However, the FDA encourages voluntary submission of such exploratory pharmacogenetic test data. In future, as more information becomes available, exploratory pharmacogenetic test data will also need to be submitted to the FDA.

Submission of pharmacogenetic data is beneficial for both the sponsor as well as the FDA. This can help in familiarizing the FDA with pharmacogenetic principles and thus reduce unnecessary delay in reviewing future submissions where pharmacogenetic testing has been used as an integral part of the drug development. Also, sponsors get to meet the FDA experts informally, discuss scientific data and get their peer opinion. The sponsor can use the pharmacogenetic test results to support the safety and efficacy of the drug compound.[46]

## Conclusion

Pharmacogenomics in pharmaceutical industry is a potential tool, awaiting use for the maximum benefit. Currently pharmacogenetic methods are being used worldwide, particularly for assessing the safety profile of drugs. Translation of the pharmacogenetic test results into clinical practice has been possible only for a small fraction of the total number of pharmacogenetic studies done. As the need for analysis of multiple genes has been realised, haplotype analysis and genome wide scan methods were designed. However, with the current cost of analysis for one SNP, haplotype analysis and genome wide scans will not enter clinical practice for testing in patients. Yet, these methods can be utilized by the pharmaceutical industry for their drug development process. Gradual inclusion of pharmacogenomic studies in drug discovery and development will cause substantial reduction in the expenses involved in drug development, ensure a safe clinical trial and reduce failures. Thus, many potential drugs which may be lost due to the effects on the outliers in a study can be retained when pharmacogenomic study is used in the future.
